# Supplementary material for: Self-reported interoceptive accuracy and interoceptive attention differentially correspond to measures of visual attention and self-regard
Source: PeerJ. 2023 May 9;11:e15348. doi: 10.7717/peerj.15348 (PMC10355190; doi:10.7717/peerj.15348)
Supplement: Supplemental Information 1 [file peerj-11-15348-s001.docx]

| Appendix A: Full correlation matrix for all variables | | | | | | | | | | | | | | | | |
| --- | --- | --- | --- | --- | --- | --- | --- | --- | --- | --- | --- | --- | --- | --- | --- | --- |
|  | Scale | M (SD) | 1 | 2 | 3 | 4 | 5 | 6 | 7 | 8 | 9 | 10 | 11 | 12 | 13 | 14 |
| 1 | IAS | 84.46 (10.94) | — |  |  |  |  |  |  |  |  |  |  |  |  |  |
| 2 | MAIA | 3.19 (0.81) | .539^***^ | — |  |  |  |  |  |  |  |  |  |  |  |  |
| 3 | MAIA-g^a^ | 3.29 (0.65) | .506^***^ | .698^***^ | — |  |  |  |  |  |  |  |  |  |  |  |
| 4 | RSES | 19.96 (2.17) | .266^**^ | .068 | .161 | — |  |  |  |  |  |  |  |  |  |  |
| 5 | FSIS^b^ | 4.61 (1.38) | .262^**^ | .094 | .438^***^ | .224^*^ | — |  |  |  |  |  |  |  |  |  |
| 6 | SWLS | 21.30 (7.51) | .316^**^ | .108 | .331^***^ | .161 | .524^***^ | — |  |  |  |  |  |  |  |  |
| 7 | DASS-D | 13.96 (6.2) | -.206^*^ | -.191 | -.242^*^ | -.299^**^ | -.434^***^ | -.123 | — |  |  |  |  |  |  |  |
| 8 | DASS-A | 13.74 (6.15) | -.219^*^ | -.167 | -.101 | -.368^***^ | -.246^*^ | .032 | .852^***^ | — |  |  |  |  |  |  |
| 9 | DASS-S | 14.79 (6.00) | -.232^*^ | -.143 | -.157 | -.304^**^ | -.259^**^ | -.038 | .848^***^ | .907^***^ | — |  |  |  |  |  |
| 10 | IAT | 0.22 (0.51) | .232^*^ | .181 | .252^*^ | .207^*^ | .235^*^ | -.045 | -.496^***^ | -.468^***^ | -.49^***^ | — |  |  |  |  |
| 11 | Flanker Acc | 0.72 (0.24) | .31^**^ | .259^*^ | .143 | .253^*^ | .094 | -.069 | -.682^***^ | -.756^***^ | -.69^***^ | .435^***^ | — |  |  |  |
| 12 | Flanker RT | 505.64 (231.70) | .213^*^ | .034 | .021 | .189 | .186 | .095 | -.476^***^ | -.404^***^ | -.431^***^ | .272^**^ | .294^**^ | — |  |  |
| 13 | Flanker Acc (diff) | -0.02 (0.316) | .128 | -.037 | -.051 | .139 | .105 | -.088 | -.368^***^ | -.451^***^ | -.407^***^ | .237^*^ | .342^***^ | .727^***^ | — |  |
| 14 | Flanker RT (diff) | 33.06 (318.14) | .222^*^ | .136 | .095 | .045 | .088 | -.096 | -.437^***^ | -.491^***^ | -.419^***^ | .195 | .619^***^ | .159 | .379^***^ | — |
| *Notes:* IAS = Interoception Awareness Scale; MAIA = Multidimensional Assessment of Interoceptive Awareness– Noticing Subscale; MAIA-g = the “general” factor of the MAIA (see text); RSES = Rosenberg Self-Esteem Scale; FSIS = Flush Self-Image Scale; SWLS = Satisfaction With Life Scale; DASS-D, DASS-A, and DASS-S = Depression Anxiety and Stress Scale and same-named subscales; IAT = *D*-scores of the self-esteem Implicit Association Task; Flanker Overall RT = RT (ms)for the whole task; Flanker Overall Acc = Proportion of correct answers for the whole flanker task; Flanker Diff. RT = Difference of RT (ms) no-flanker blocks from flanker trials in mixed blocks; Flanker Diff. Acc. = Difference of proportion of correct answers on no-flanker blocks from flanker trials in mixed blocks;  ^a^The average response across six (of eight) subscales on the MAIA (see text)  ^b^The mean distance from negative attribute to positive attribute on a scale of 0–7;  ^*^ *p* < .05; ^**^ *p* < .01; ^***^ *p* < .001. | | | | | | | | | | | | | | | | |
