# Supplementary material for: Self-reported interoceptive accuracy and interoceptive attention differentially correspond to measures of visual attention and self-regard
Source: PeerJ. 2023 May 9;11:e15348. doi: 10.7717/peerj.15348 (PMC10355190; doi:10.7717/peerj.15348)
Supplement: Supplemental Information 2 [file peerj-11-15348-s002.docx]

Appendix B: Progression of component structure used in the current study

**Appendix B.1: Unrotated factor solution**

|  | **1** | **2** | **3** | **Uniqueness** |
| --- | --- | --- | --- | --- |
| DASS-A | -.919 |  |  | .120 |
| DASS-D | -.902 |  |  | .154 |
| DASS-S | -.896 |  |  | .176 |
| Flanker Overall Acc | .804 |  |  | .217 |
| IAT-D | .592 |  |  | .628 |
| Flanker Diff. Acc | .578 |  |  | .526 |
| RSES | .400 |  |  | .724 |
| SWLS |  | .845 |  | .281 |
| FSIS |  | .756 |  | .290 |
| Flanker Overall RT | .594 |  | .726 | .118 |
| Flanker Diff. RT | .596 |  | .690 | .128 |

**Appendix B.2: Promax rotation**

|  | **1** | **2** | **3** | **Uniqueness** |
| --- | --- | --- | --- | --- |
| Flanker Overall RT | -.939 |  |  | .217 |
| DASS-A | .918 |  |  | .120 |
| DASS-S | .867 |  |  | .176 |
| DASS-D | .827 |  |  | .154 |
| Flanker Diff. Acc | -.687 |  |  | .526 |
| IAT-D | -.602 |  |  | .628 |
| SWLS |  | .864 |  | .281 |
| FSIS |  | .805 |  | .290 |
| RSES |  |  |  | .724 |
| Flanker Overall RT |  |  | .930 | .118 |
| Flanker Diff. RT |  |  | .912 | .128 |

**Appendix B.3: Varimax rotation**

|  | | **1** | **2** | **3** | **Uniqueness** |
| --- | --- | --- | --- | --- | --- |
| DASS-A | | -.896 |  |  | .120 |
| Flanker Overall RT | | .877 |  |  | .217 |
| DASS-S | | -.852 |  |  | .176 |
| DASS-D | | -.821 |  |  | .154 |
| Flanker Diff. Acc | | .650 |  |  | .526 |
| IAT-D | | .578 |  |  | .628 |
| Flanker Overall RT |  | | .901 |  | .118 |
| Flanker Diff. RT | |  | .890 |  | .128 |
| SWLS | |  |  | .835 | .281 |
| FSIS | |  |  | .823 | .29 |
| RSES | |  |  | .416 | .724 |

**Appendix B.4: Final structure used in the current study**

|  | **1 (*signs and symptoms*)** | **2 (*self-regard*)** | **Uniqueness** |
| --- | --- | --- | --- |
| DASS-A | -.908 |  | .148 |
| DASS-S | -.868 |  | .197 |
| Flanker Overall Acc | .841 |  | .293 |
| DASS-D | -.836 |  | .172 |
| Flanker Diff. Acc | .648 |  | .551 |
| Flanker Diff. RT | .628 |  | .604 |
| IAT-D | .566 |  | .650 |
| Flanker Overall RT | .560 |  | .645 |
| SWLS |  | .831 | .283 |
| FSIS |  | .828 | .290 |
| RSES |  | .425 | .731 |

*Notes*: Only factor loadings ≥ .4 are shown; IAS = Interoception Awareness Scale; RSES = Rosenberg Self-Esteem Scale; FSIS = Flush Self-Image Scale; SWLS = Satisfaction With Life Scale; DASS-D, DASS-A, and DASS-S = Depression Anxiety and Stress Scale and same-named subscales; IAT-D = *D*-scores of the self-esteem Implicit Association Task; Flanker Overall RT = Reaction Time for the whole task; Flanker Overall Acc = Proportion of correct answers for the whole flanker task; Flanker Diff. RT = difference of RT no-flanker blocks from flanker trials in mixed blocks (no-flanker – flanker); Flanker Diff. Acc. = Difference of proportion of correct answers on no-flanker blocks from flanker trials in mixed blocks (no-flanker – flanker);
